# Co-expression network analysis identifies potential candidate hub genes in severe influenza patients needing invasive mechanical ventilation

**DOI:** 10.1186/s12864-022-08915-9

**Published:** 2022-10-15

**Authors:** Liang Chen, Jie Hua, Xiaopu He

**Affiliations:** 1 Department of Infectious Diseases, Nanjing Lishui People’s Hospital, Zhongda Hospital Lishui Branch, Southeast University, Nanjing, China; 2 Department of Gastroenterology, Liyang People’s Hospital, Liyang Branch Hospital of Jiangsu Province Hospital, Nanjing, China; 3 Department of Geriatric Gastroenterology, The First Affiliated Hospital With Nanjing Medical University, No.300 Guangzhou Road, Nanjing city, 210029 Jiangsu Province China

**Keywords:** Severe influenza, Invasive mechanical ventilation, Co-expression network analysis, Hub gene

## Abstract

**Background:**

Influenza is a contagious disease that affects people of all ages and is linked to considerable mortality during epidemics and occasional outbreaks. Moreover, effective immunological biomarkers are needed for elucidating aetiology and preventing and treating severe influenza. Herein, we aimed to evaluate the key genes linked with the disease severity in influenza patients needing invasive mechanical ventilation (IMV). Three gene microarray data sets (GSE101702, GSE21802, and GSE111368) from blood samples of influenza patients were made available by the Gene Expression Omnibus (GEO) database. The GSE101702 and GSE21802 data sets were combined to create the training set. Hub indicators for IMV patients with severe influenza were determined using differential expression analysis and Weighted correlation network analysis (WGCNA) from the training set. The receiver operating characteristic curve (ROC) was also used to evaluate the hub genes from the test set's diagnostic accuracy. Different immune cells' infiltration levels in the expression profile and their correlation with hub gene markers were examined using single-sample gene set enrichment analysis (ssGSEA).

**Results:**

In the present study, we evaluated a total of 447 differential genes. WGCNA identified eight co-expression modules, with the red module having the strongest correlation with IMV patients. Differential genes were combined to obtain 3 hub genes (HLA-DPA1, HLA-DRB3, and CECR1). The identified genes were investigated as potential indicators for patients with severe influenza who required IMV using the least absolute shrinkage and selection operator (LASSO) approach. The ROC showed the diagnostic value of the three hub genes in determining the severity of influenza. Using ssGSEA, it has been revealed that the expression of key genes was negatively correlated with neutrophil activation and positively associated with adaptive cellular immune response.

**Conclusion:**

We evaluated three novel hub genes that could be linked to the immunopathological mechanism of severe influenza patients who require IMV treatment and could be used as potential biomarkers for severe influenza prevention and treatment.

## Background

Influenza is a contagious respiratory disease caused by the influenza virus. Despite advances in medical technology, influenza continues to cause many hospitalizations and deaths [1, 2]. Symptomatic influenza affects 10–20% of the global population yearly, with 3–5 million cases of severe disease and 290,000–650,000 deaths reported [3]. Furthermore, influenza is a disease with a wide range of clinical manifestations, from a self-limited upper respiratory tract infection to severe pneumonia [4, 5]. With a mortality rate as high as 50–80%, critically ill patients with influenza frequently have severe respiratory failure (e.g., arterial pressure of oxygen/fraction of inspiration oxygen 200 mmHg) and require invasive mechanical ventilation (IMV) [6–8]. Moreover, the detailed pathogenesis of severe influenza is still unknown.

Previous studies have revealed that immunological cells and immune pathways have been implicated in the onset and progression of severe influenza [9, 10]. Hence, it is needed to evaluate promising immunological biomarkers for diagnosing and treating patients with severe influenza. Bioinformatics analysis has been frequently used to screen disease-specific biomarkers since the emergence of microarray technologies [11, 12]. However, it is challenging to conduct statistical evaluations and extract relevant information due to sample heterogeneity and sampling discrepancies, different technology platforms, and technique utilization in individual studies [13–15]. Hence, integrated bioinformatics tools that provide comprehensive and valuable information have been employed to investigate the molecular pathophysiology of influenza infection and evaluate novel biomarkers. Weighted correlation network analysis, also known as weighted gene co-expression network analysis (WGCNA), is a tool utilized for indicating gene interaction patterns across models, which can characterize the relationship between genes and interaction pathways based on the endogeneity of the gene set and the link between the gene set and the phenotype [16, 17]. Therefore, this promising method is increasingly being utilized to identify highly synergistic gene sets, potential biomarker genes, and therapeutic targets.

Herein, we aimed to evaluate the key genes linked with the severe influenza patients needing invasive mechanical ventilation (IMV) by WGCNA and their association with the infiltration levels of distinctive immune cells via single-sample gene set enrichment analysis (ssGSEA).

## Results

### The stable DEGs detection and functional enrichment between IMV and NIMV group

**Fig. 1:**
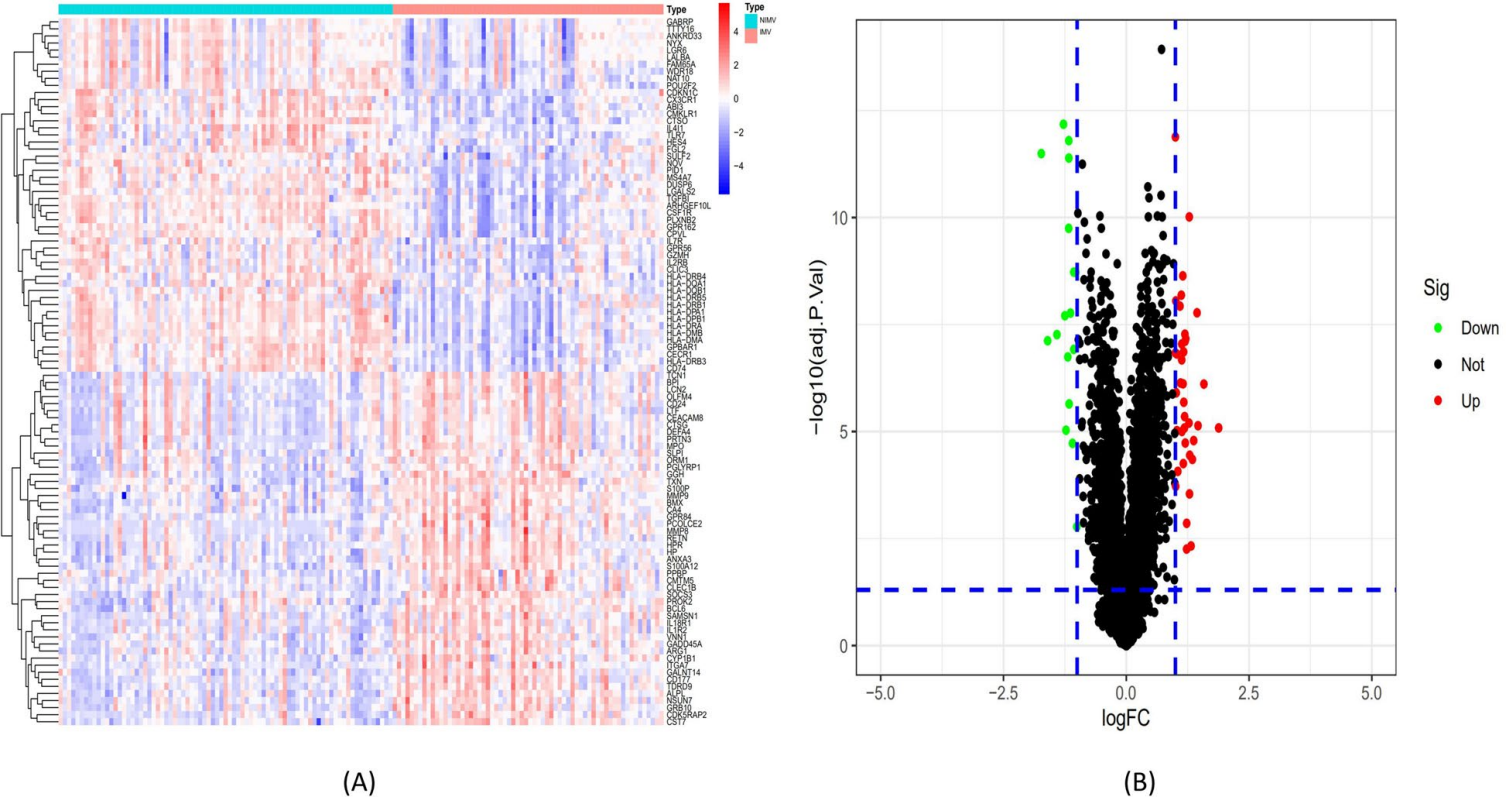
Illustrates the training set gene expression profiling. **A** A heatmap of the top 50 DEGs. Upregulated genes are seen in red, while downregulated genes are highlighted in blue. **B** The DEGs volcanic plot. Upregulated genes are highlighted in red, while downregulated genes are indicated in green

Differentiation analysis was employed to examine the gene expression profiling from the training set. According to the criteria, between IMV and NIMV patients with influenza, a total of 447 DEGs (containing 261 upregulated and 186 downregulated genes) were found (Fig. 1).

**Fig. 2:**
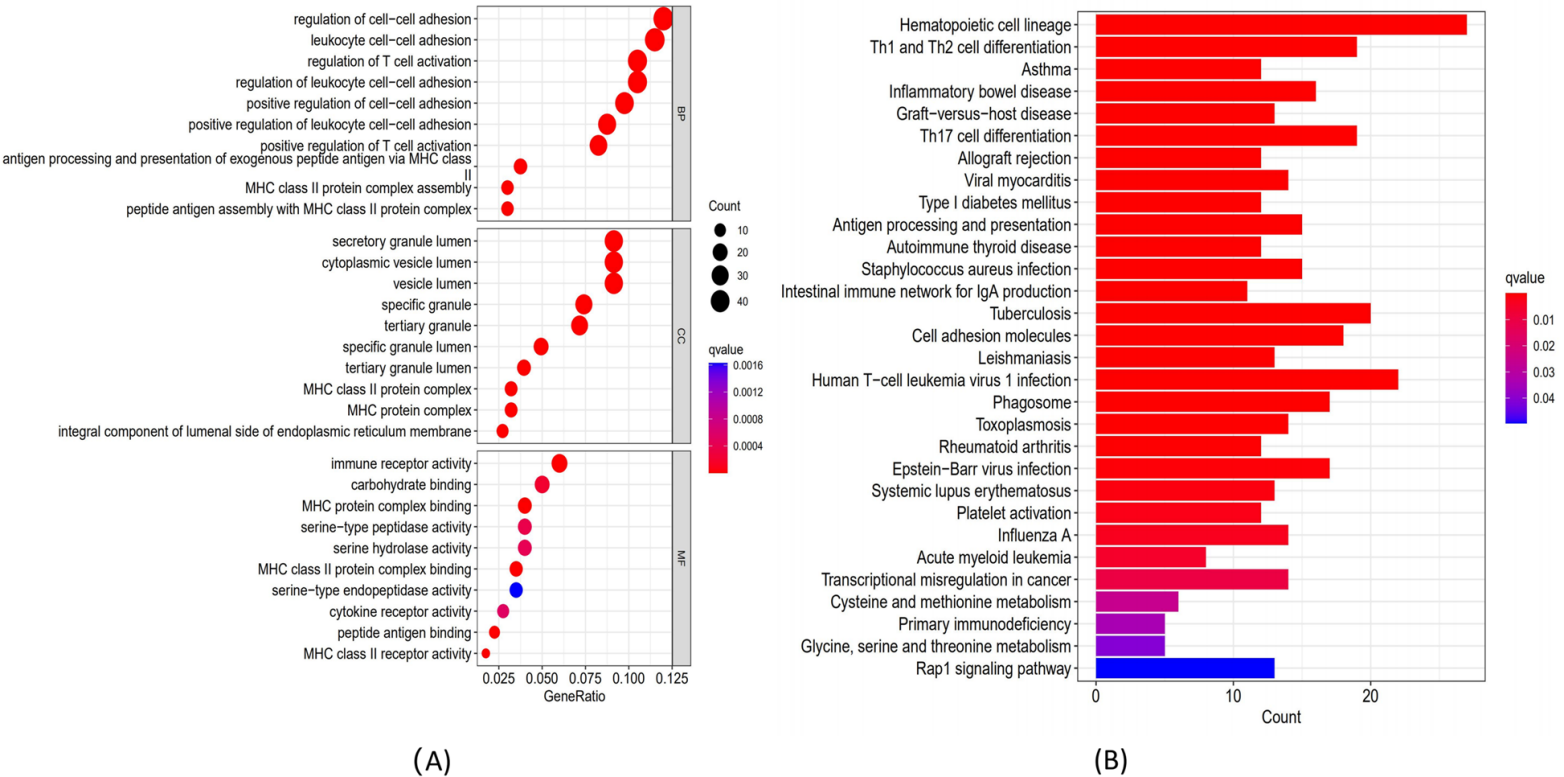
The DEGs from the training set were analyzed for functional enrichment Analysis of; **A** GO enrichment, and **B** Analysis of KEGG enrichment

The biological activities and signal cascades of DEGs co-related with severe influenza patients with IMV were studied using GO and KEGG analyses. Based on the GO enrichment analysis, DEGs were mostly involved in antigen processing and presentation (e.g., antigen processing and presentation of exogenous peptide antigen via MHC class II, MHC class II protein complex assembly, and peptide antigen assembly with MHC class II protein complex) and cell–cell adhesion (e.g., regulation of T cell activation), as shown in Fig. 2A. According to the KEGG signaling cascades analyses, DEGs were abundant in pathways linked to infection and cellular differentiation (e.g., Th1, Th2, and Th17 cell differentiation, *Staphylococcus aureus* infection, hematopoietic cell lineage, and human T-cell leukaemia virus-1 infection) (Fig. 2B). These findings demonstrated the immunological and inflammation-related pathways are associated with influenza severity in patients requiring IMV for severe influenza.

### WGCNA and hub gene analysis

**Fig. 3:**
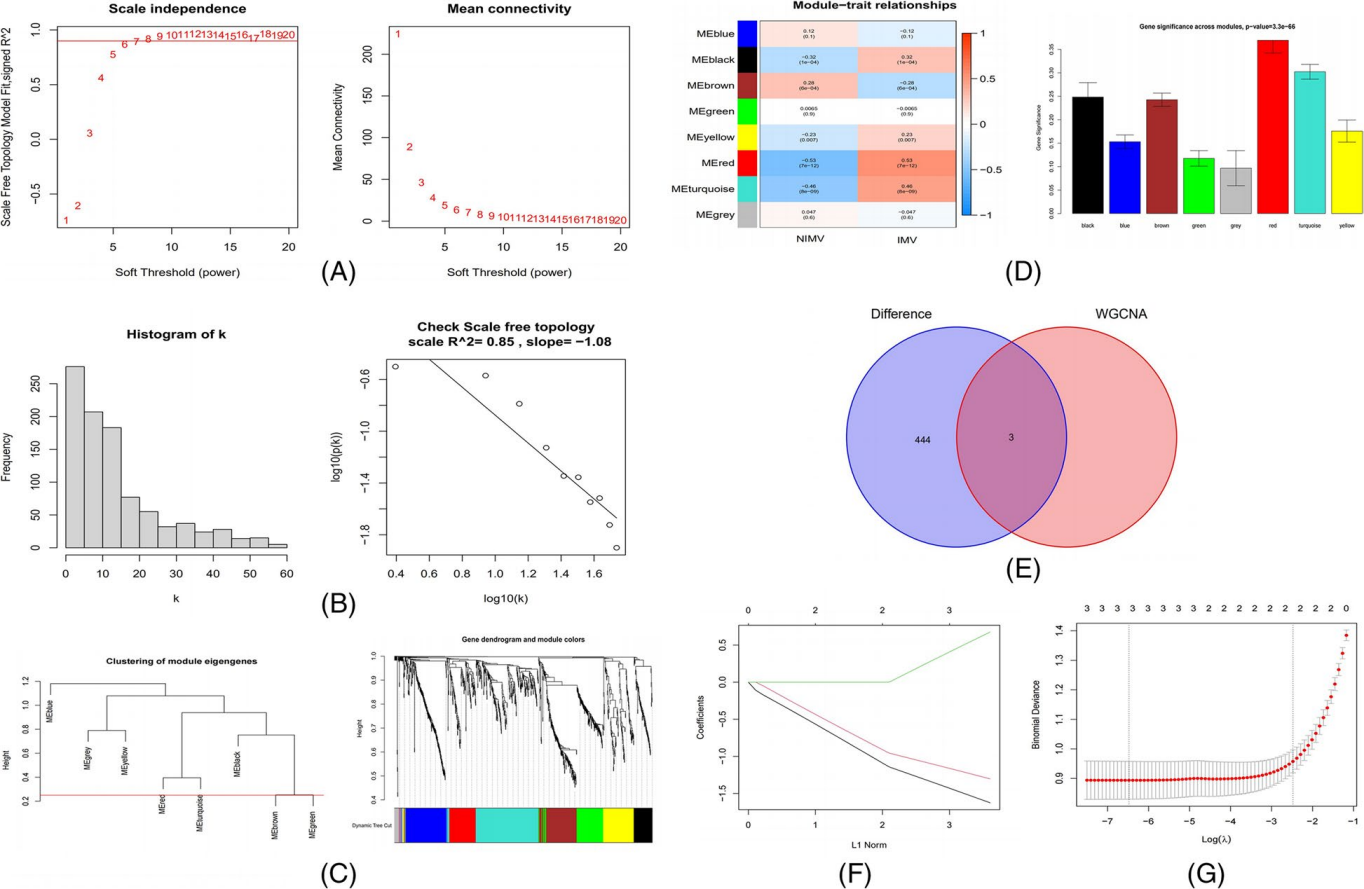
Establishing a weighted gene co-expression network analysis (WGCNA) and screening for Hub genes. **A** Scale-free fit index and mean connectivity analysis for various soft-thresholding powers. The red line denotes the point when the correlation coefficient is 0.9, and the soft-thresholding power (β) is 6. **B** Connectivity distribution histogram and scale-free topology check when β = 6. **C** Gene dendrograms and clustering of module eigengenes using a dissimilarity metric (1-TOM) (the red line represents a cut height of 0.25). **D** Module-trait correlations between module eigengenes and sample traits were assessed. The correlation coefficient and *P* value are displayed in each cell. **E** Venn diagram showing where the DEGs and the red module overlap. **F** LASSO regression's partial likelihood deviance with changing log_(l)_ is shown in tenfold cross-validations. Using the minimum criterion (lambda.min) and 1 standard error of the minimum criterion (1-SE criteria), dotted vertical lines were created at the ideal values. **G** The tenfold cross-LASSO validation coefficient profiles for three hub genes are shown

The training set was utilized to build the co-expression network by WGCNA analysis to evaluate the important modules linked with influenza severity. The significance of the diagnosis and evaluation of hub gene expression level was examined using a soft-thresholding power of 6 (scale-free *R*^2^ = 0.85) and a cut height of 0.25. We then narrowed it down to eight modules, as shown in Fig. 3A-C. The link between module eigengene (ME) values and sample traits was used to quantify the link between the modules and clinical sample traits, which was observed using heat map profiling. The red module was the most closely related to illness severity (cor = 0.53, *P* = 7e-12) (Fig. 3D). Subsequently, three intersecting genes were determined based on DEG intersections (Fig. 3E). LASSO analysis confirmed 3 hub genes as follows: CECR1, HLA-DPA1, and HLA-DRB3 (Fig. 3F-G).

### Evaluation of hub gene expression and diagnostic value

**Fig. 4:**
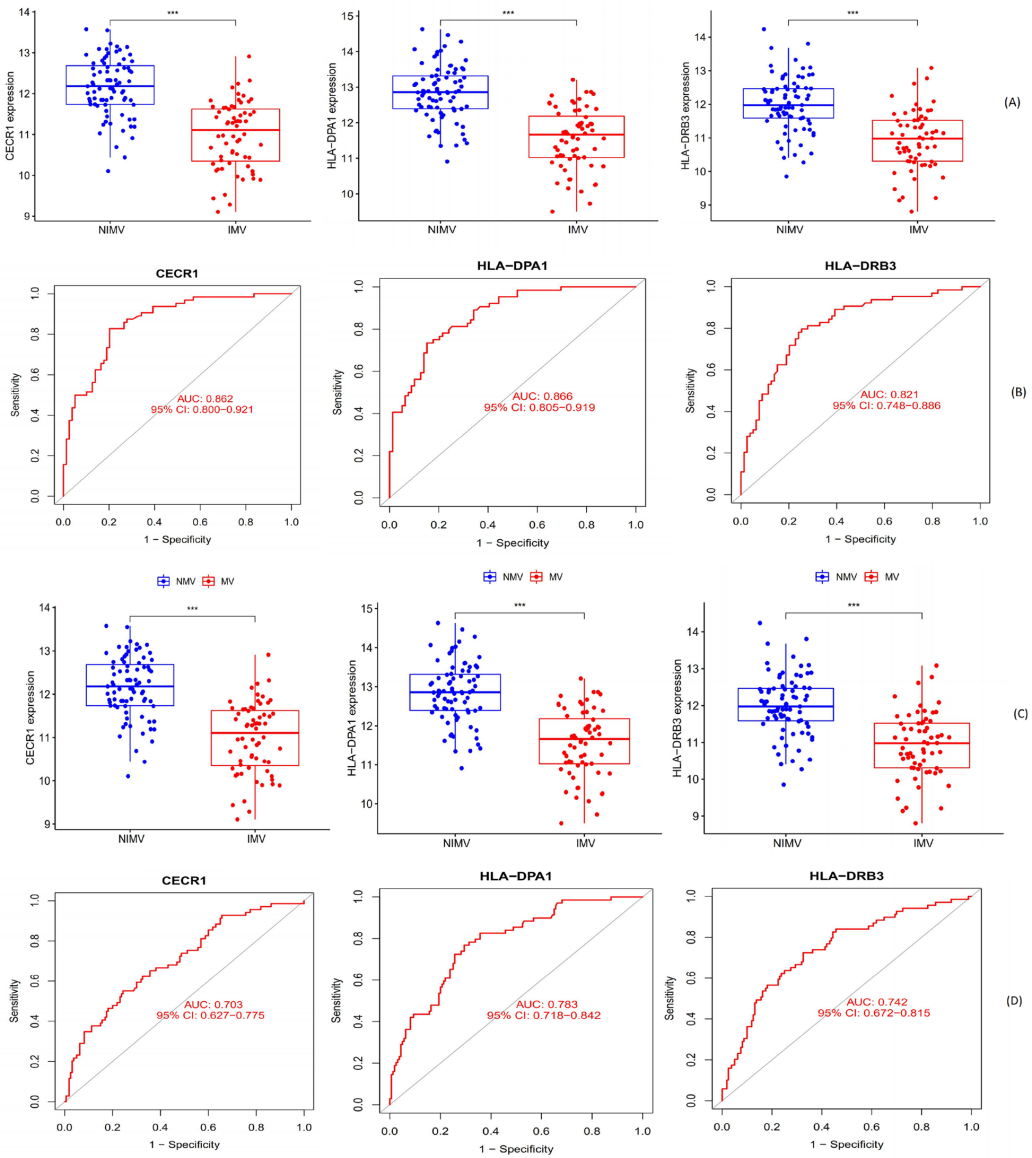
Confirmation of hub genes. **A** Boxplots in the training set were applied to validate the hub genes' expression levels. **B** ROC analysis in the training set was used to validate the diagnostic utility of the hub genes. **C** Boxplots in the test set were used to verify the hub genes' expression levels, and (**D**) ROC analysis in the test set was utilized to establish the hub genes' diagnostic value

Boxplots were utilized to identify the expression of the three hub genes. Figure 4A shows that IMV patients have considerably lower CECR1, HLA-DPA1, and HLA-DRB3 expression levels than NIMV patients from the training set. Moreover, similar results were observed in the patients from the test set (Fig. 4C). ROC analysis from the training set showed that area under the ROC curve (AUC) was 0.862 [*95% confidence interval (CI)* 0.800–0.921] for CECR1, 0.866 (*95% CI* 0.805–0.919) for HLA-DPA1, and 0.821 (*95% CI* 0.748–0.886) for HLA-DRB3, accordingly (Fig. 4B). Meanwhile, the AUC was 0.700–0.800 for the three hub genes from the test set, suggesting a moderate diagnostic accuracy (Fig. 4D).

### The link between hub genes and ICI

**Fig. 5:**
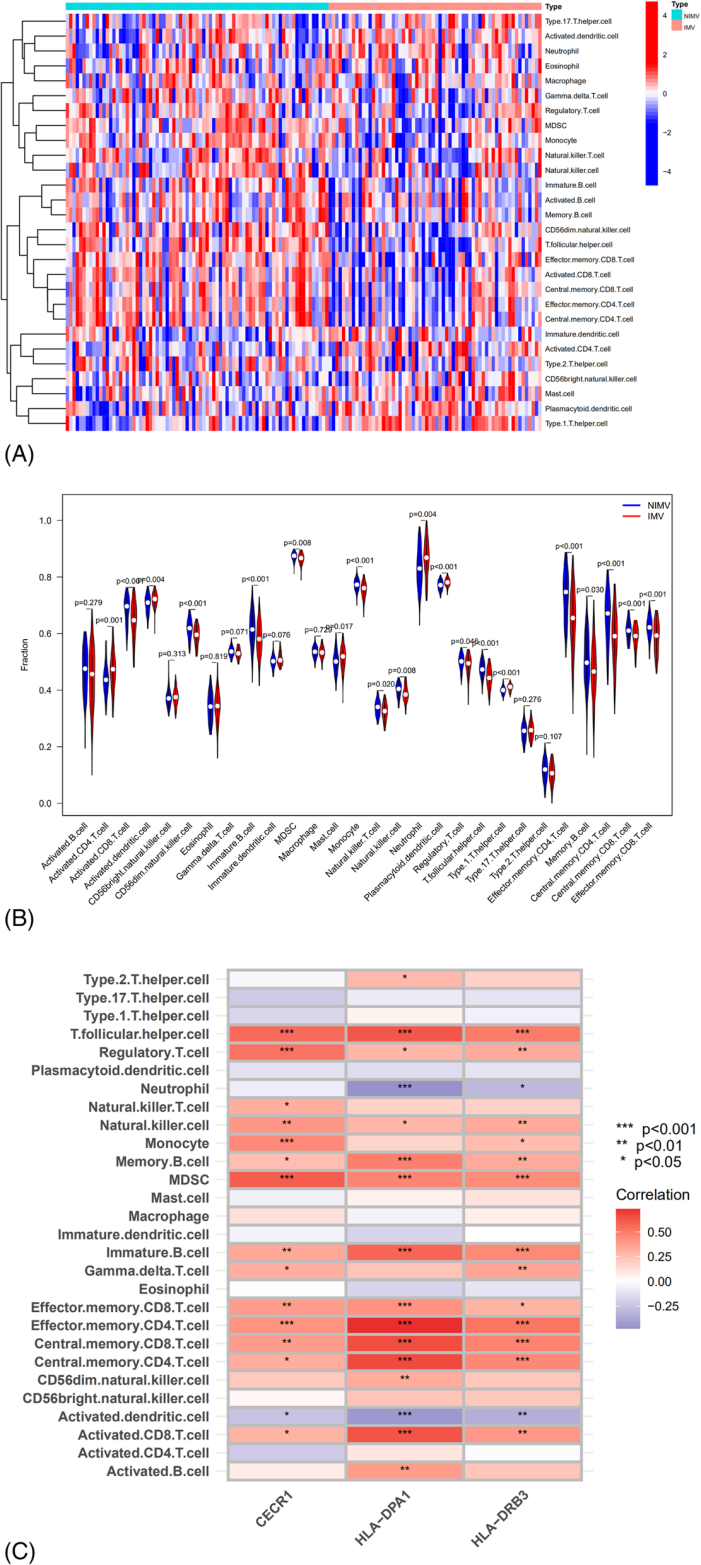
Analysis of the immunological landscape concerning disease severity. The distribution of immune cells in the IMV and NIMV groups is depicted in an (**A**) heatmap and (**B**) violin plot. **C** The connection between immune cell infiltration and hub genes

The ssGSEA algorithm was employed to evaluate the variances in ICI between IMV and NIMV groups. The results obtained from sGSEA revealed a considerably elevated infiltration of neutrophils and dendritic cells, a lower infiltration of activated CD8 T cells, memory CD8 T cells, memory CD4 T cells, and natural killer cells in the IMV group. Immunological cell correlation analysis with hub genes suggested that the three hub genes were positively linked with activated CD8 T-cells, T follicular helper cells, memory CD8 T-cells, and memory CD4 T-cells, while negatively associated with activated dendritic cells. In addition, HLA − DPA1 and HLA − DRB3 were negatively related to neutrophils (Fig. 5A, B).

## Discussion

Influenza is a contagious respiratory disease caused by the influenza virus. Despite advances in medical technology, influenza continues to cause many hospitalizations and deaths [1–3]. In recent decades, transcriptomics studies revealed that the structure of gene sets and their roles differed throughout the broad spectrum of patients with varying degrees of severity [13–15, 18, 19]. By combining numerous datasets and employing systematic bioinformatics tools, we identified three key genes related to severe influenza patients requiring IMV. WGCNA offers numerous advantages over other bioinformatics methods because the analysis focuses on the link between clinical features and co-expression modules, resulting in more complete data with high reliability and biological significance.

In this study, the functional enrichment analysis indicated that the DEGs between IMV and NIMV patients were mostly linked with immunological and inflammatory cascades, consistent with previous findings [18–21]. Moreover, the ICI showed two opposing results. First, the adaptive cellular immune response to influenza viruses was suppressed, as evidenced by a significant drop in active and memory CD8 + T cells in IMV patients, despite an increase in activated dendritic cells and Th1 cells. Second, the neutrophils were found to be activated. A similar study by Nguyen et al. [19] reported comparable results. They revealed a relationship between increased Sequential Organ Failure Assessment (SOFA) and lower adaptive interferon (IFN)-γ producing CD8 + T cell responses in people hospitalized with acute influenza. According to Jake Dunning et al., patients with the most severe illnesses also have a higher proportion of transcripts associated with neutrophils and fewer transcripts connected to IFN- γ [21]. Although multiple studies [22, 23] have shown a correlation between lymphopenia and severe influenza, the mechanism by which the adaptive cellular immune response is inhibited in severe influenza remains largely unknown [24–26]. When the lungs get infected with influenza, neutrophils are among the first cells attracted there and perform a defensive function [27]. In severe influenza infection, higher numbers of circulating and lung neutrophils are associated with, but not dependent on, bacterial co-infection [28, 29]. A significant neutrophil influx was associated with severe lung inflammation, whereas neutrophil reduction had little effect on viral clearance and caused only minor lung pathology [27–29]. Myeloperoxidase and neutrophil extracellular traps (NETs), which have been recognized as endothelial-damaging agents, are released by neutrophils [30, 31]. NETs produced by influenza infection may compromise lung function rather than contribute to bacterial killing or protect against secondary bacterial infection. According to recent studies, neutrophil-dependent tissue damage causes mortality regardless of viral load, and DNase neutralization of NETs enhances longevity [32].

Furthermore, poor outcomes in septic patients have previously been associated with decreased expression of MHC class II-related genes, including HLA-DPA1 and HLA-DRB3 [33–35]. Several inflammatory mediators may be responsible for the downregulation of MHC II mRNA expression [36, 37]. Rare accounts, nonetheless, did concentrate on influenza in the literature. We hypothesized that decreased MHC II expression results in impaired antigen processing and presentation, abnormal lymphocyte proliferation, and impaired viral clearance, all of which affect the development of severe influenza. The CECR1 (Cat Eye Syndrome Chromosome Region 1) gene, found on chromosome 22q11, also produces the ADA2 protein. According to previous research, ADA2 deficiency is frequently linked to lymphopenia (including CD8 memory cells, T cells, and follicular T cells) [38, 39]. It is also linked to increased myeloperoxidase expression and up-regulation of neutrophil-expressed gene transcripts, which can lead to the formation of NET [40]. In severe influenza, these could cause endothelium damage and severe illness. The current study indicated that the expression of three key genes was considerably lower in severe influenza patients requiring IMV compared to NIMV patients.

This study has some limitations. First, the sample size is still relatively small, which might limit the accuracy of the obtained data. Second, we should classify the association of hub genes and immune cells identified in this study as just a statistical correlation rather than causality. Finally, despite identifying several DEGs in IMV and NIMV patients, it is unknown if these host factors are unique to severe influenza infection. Hence, in vivo and in vitro studies are necessary to identify the function of identified hub genes and the underlying mechanism in severe influenza.

## Conclusions

In conclusion, we evaluated three novel hub genes that could be linked to the immunopathological mechanism of severe influenza patients that require IMV treatment and could be used as candidate biomarkers for severe influenza prevention and treatment.

## Methods

### Data source

The NCBI-GEO (http://www.ncbi.nlm.nih.gov/geo) was used to obtain the mRNA expression patterns of influenza patients' blood samples. The selection criteria included: i) influenza infection confirmed by RT-PCR using respiratory tract samples; ii) identical disease severity categorization; and iii) influenza patients ≥ 16 years of age and intubated. In this study, we identified three data sets such as GSE101702, GSE21802, and GSE111368. The GSE21802 microarray data contained 20 whole-blood samples from IMV patients and 16 whole-blood samples from non-IMV patients (NIMV), whereas the GSE101702 included 44 whole-blood samples from the IMV group and 63 whole-blood samples from the NIMV group. The microarray data of GSE111368 included 69 whole-blood samples from the IMV group and 160 whole-blood samples from the NIMV group. The training data for the GSE101702 and GSE21802 were combined to identify differentially expressed genes (DEGs) to construct WGCNA. The hub genes were validated using GSE111368 as the test set.

### Evaluation of DEGs

The 'limma' and 'GEOquery' packages of R software (version 4.2.0) were used to normalize and annotate the data from the training set (GSE101702 + GSE21802), with DEG screening criteria of adjusted *P*-value < 0.05 and log fold change (logFC) > 0.5. The data was plotted on a volcano, and the top 50 DEGs were plotted on a heatmap.

### Functional enrichment analysis

The R package 'cluster-profile' was utilized to conduct GO enrichment and KEGG pathway analyses. With an adjusted *P* < 0.05, GO terms or KEGG cascades were considered statistically significant. Biological process, cellular component, and molecular function (abbreviated as BP, CC, and MF, respectively) were GO terms' three aspects.

### Generation of WGCNA

With the 'WGCNA' package of R software, a WGCNA was generated for the expression profile data of the training set. Next, genes were selected with the top 25% absolute deviation from the median. The 'goodSampleGenes' function was used to ensure that the data was accurate. With the help of the 'pickSoftThreshold' function, an ideal soft threshold (β) was chosen and confirmed. The data from the matrix were then changed into an adjacency matrix, clustered to find modules based on the topological overlap. A hierarchical clustering dendrogram was constructed after completing the module eigengene (ME) calculation and combining related modules in the ME-based clustering tree. Gene significance (GS) and module significance (MS) were evaluated using modules and phenotypic data to identify the GS and clinical information and examine the relationship between modules and models. In addition, for each gene, the module membership (MM) was determined to examine the GS in the module.

### Hub gene evaluation and confirmation

Candidate hub genes were chosen based on their significant inter-module interaction. The absolute GS values of genes having biological importance are frequently greater. The criteria were used to screen candidate hub genes (absolute value of GS > 0.20; absolute value of MM > 0.80). The DEGs were then intersected with the potential hub genes using the R software's 'glmnet' package to conduct LASSO analysis to find the final hub genes.

Box plots were used to compare the levels of hub gene expression in the IMV and NIMV groups. The expression of hub genes was assessed through receiver operating characteristic curves (ROCs) to differentiate IMV from NIMV patients.

### Evaluation of immune cell infiltration (ICI) and its association with hub genes

The ssGSEA algorithm was used to calculate the relative infiltration levels of immune cells in the training set samples. Differential expression levels of immune invading cells were visualized using violin plots. The 'ggplot2' package was used to visualize the Spearman correlations for immune infiltrating cells with hub genes.

## Data Availability

Publicly available datasets were analyzed in this study. These data can be found in GSE101702 (https://www.ncbi.nlm.nih.gov/geo/query/acc.cgi?acc=GSE101702), GSE21802 (https://www.ncbi.nlm.nih.gov/geo/query/acc.cgi?acc=GSE21802) and GSE111368 (https://www.ncbi.nlm.nih.gov/geo/query/acc.cgi?acc=GSE111368).

## Abbreviations

IMV: Invasive mechanical ventilation
NIMV: Noninvasive mechanical ventilation
GEO: Gene Expression Omnibus
ROC: Receiver operating characteristic curve
SsGSEA: Single-sample gene set enrichment analysis
WGCNA: Weighted gene co-expression network analysis
LASSO: Least absolute shrinkage and selection operator
DEGs: Differentially expressed genes
SOFA: Sequential Organ Failure Assessment
CI: Confidence interval
